# Assessment of Forward Osmosis in PRO Mode during Desalination of a Local Oil Refinery Effluent

**DOI:** 10.3390/membranes11110801

**Published:** 2021-10-21

**Authors:** Elorm Obotey Ezugbe, Emmanuel Kweinor Tetteh, Sudesh Rathilal, Dennis Asante-Sackey

**Affiliations:** Green Engineering and Sustainability Research Group, Department of Chemical Engineering, Faculty of Engineering and The Built Environment, Durban University of Technology, Durban 4001, South Africa; elormezugbe.ee6@gmail.com (E.O.E.); rathilals@dut.ac.za (S.R.); ingsackey@gmail.com (D.A.-S.)

**Keywords:** desalination, forward osmosis, fouling, membrane orientation

## Abstract

In this study, the performance of a forward osmosis system was assessed over a 30-h period during desalination of a local oil refinery effluent using NaCl as the draw solute. The study was conducted with the active layer of the membrane facing the draw solution. Assessment was done based on the water flux, salt rejection (SO_4_^2−^ and CO_3_^2−^), membrane fouling and fouling reversal after membrane cleaning. Critical to this study was the performance of manual scrubbing of the membrane after each run and the application of chemically enhanced osmotic backwash. Scanning electron microscope (SEM) analysis of the cellulose triacetate (CTA) membrane was conducted before and after cleaning to ascertain the degree of fouling and fouling reversal after membrane cleaning. The results showed an average water flux of 3.78 ± 0.13 L/m^2^ h, reverse solute flux (RSF) of 1.56 ± 0.11 g/m^2^·h, SO_4_^2−^ rejection of 100%, CO_3_^2−^ rejection of 95.66 ± 0.32% and flux recovery of 95% after membrane cleaning. This study identifies that intermittent manual scrubbing of the membrane plays a major role in overall membrane performance. It also provides a practical basis for further research and decision making in the use of FO and CTA membranes for oil refinery effluent desalination.

## 1. Introduction

Oil refineries use large volumes of water in processing crude oil to finished products. Consequently, huge amounts of effluents are generated which are heavily laden with all forms of contaminants. By virtue of the sources of these effluents, their composition is highly complex and inconsistent. This highly contaminated water is made up of high concentrations of residual free and emulsified oils, hydrocarbons (representing the main organic load), dissolved salts (halides, phosphates, sulphates and sulfides) and carcinogenic and mutagenic substances which require rigorous treatment for their proper removal [1]. Dissolved salts form a major part of oil refinery effluents (ORE), having been noted to have concentrations of up to 35,000 mg/L and posing treatment challenges to conventional effluent treatment facilities [2].

Over recent decades, desalination applications saw a paradigm shift from thermal treatment processes to membrane-based processes, specifically reverse osmosis (RO) [3]. While RO has proven to be cheaper and more convenient than conventional thermal desalination processes, concerns of high energy consumption due to the pressure requirements, high fouling propensity of the membranes and its dependence on chemicals have been raised. Ever since, alternative desalination methods such as FO, membrane distillation, electrodialysis, etc., are being explored fervently [3,4].

Against this background, forward osmosis (FO) research emerges as a prospective solution to water scarcity issues, looking to significantly complement the drawbacks of pressure-driven membrane processes [5]. Technically, the dependence of FO on osmotic pressure gradient for separation and water transport across a semi-permeable membrane presents many explorable options for membrane fouling control and low-pressure separation [6]. This is in contrast with principles of pressure-driven membrane processes such as reverse osmosis (RO) and nano filtration (NF), which depend on external hydraulic pressure for mass transport across a semi-permeable membrane. Consequently, the application of FO can lead to the reduction of capital, energy and operating costs [7]. This has been demonstrated by several studies in sea and brackish water desalination [8,9], water reclamation and wastewater treatment [10,11].

A significant part of FO research has focused mainly on membrane development and improvement aimed at reducing internal concentration polarization (ICP) and improving hydrophilicity [12,13,14]. Additionally, recent research has looked at draw solute development aimed at generating high osmotic pressure to enhance water flux across the membrane, while ensuring that it is easily recovered from water [15,16]. Other forms of FO research have paid attention to the optimization of FO operating parameters and the hydrodynamics of fluid flow through feed and draw solution channels aimed at improving boundary layer conditions, reducing reverse solute diffusion (RSD) and membrane fouling [17,18,19].

In FO applications, membrane orientation plays a vital role in carrying out the process. FO membranes are usually asymmetric, made of a dense active layer and a porous support layer. As such, the performance of the process is completely different when the feed solution (FS) is placed against either of the two different sides of the membrane [20]. When the FS is placed against the active layer, it is referred to as the FO mode whereas the FS against the support layer is referred to as the PRO mode. Typical of the membrane orientations are the effects of concentration polarization (CP). CP occurs when particle concentration at the membrane surface is higher than in the bulk of the fluid, known as external concentration polarization (ECP). When CP occurs within the porous support layer of the membrane, it is known as internal concentration polarization (ICP) [21]. ICP may be dilutive (dilutive internal concentration polarization (DICP)) or concentrative (concentrative internal concentration polarization (CICP)), depending on the side of the membrane that faces the FS. DICP occurs in the FO mode whereas CICP occurs in the PRO mode. ICP is highly undesirable; it causes a significant reduction in the osmotic pressure difference between the bulk FS and the bulk DS. Consequently, the net driving force of the process is reduced [5,22].

Several studies have been conducted to investigate the effects of membrane orientation on FO applications. Zhao, et al. [23] compared the performances of FO in both PRO and FO modes. The authors observed that the choice of membrane orientation is highly dependent on the feed solution concentration and the degree of concentration expected. Additionally, the authors concluded that the FO mode produced higher and more stable flux with higher flux recovery after membrane cleaning. In a similar study, Wang, et al. [24] used latex particles as model foulants to investigate the effects of membrane orientation on fouling resistance. The authors observed that while nearly 70% of the membrane surface was covered by the latex particles during the PRO mode, coverage in the FO mode was nearly zero. This was attributed to the smooth nature of the active layer and the low flux levels. It was then concluded that the FO mode showed more resistance to fouling compared to the PRO mode. Table 1 shows some examples of CTA membranes and their applications in different orientations.

In a study conducted by Honda, et al. [30], using a CTA membrane, it was established that the PRO mode of FO operation is characterised by high initial water fluxes and rapid flux declines. This was attributed to the surface characteristics of the support layer, in terms of surface roughness and loose pores. Rougher support layers have been reported to promote foulant attachment and subsequent diffusion into the membrane pores [31,32]. Similar observations were made by Xie, et al. [33], where the authors established through their work that water flux in the PRO mode was considerably higher than in the FO mode. The authors concluded that the observed difference was due to the effects of ICP. Detailed explanation of the effects of ICP on flux decline has been given in previous studies [20,34]. That is, the possibility of obtaining high fluxes in the PRO mode and maintaining the fluxes while achieving excellent salt rejection efficiencies is high and gives room for exploration.

This study therefore assessed the performance of a forward osmosis system over a period of 30 h during desalination of a local oil refinery effluent using NaCl as draw solute. The study was conducted with the active layer of the membrane facing the draw solution (PRO mode) using a CTA membrane sourced from Fluid Technology Solutions, OR, USA. Assessment was done based on the permeation flux, salt rejection efficiency (SO_4_^2−^ and CO_3_^2−^), membrane fouling and fouling reversal after membrane cleaning. An important part of the current study was the performance of manual scrubbing of the membrane after each run, the application of chemically enhanced osmotic backwash and how they impacted on the overall performance of the process. SEM analysis of the CTA membrane was conducted before and after cleaning to ascertain the degree of fouling and fouling reversal after membrane cleaning.

## 2. Materials and Methods

### 2.1. Experimental Set-Up and CTA Membrane

The set up used in this study is similar to the one depicted in our previous study [11]. Flat sheet cellulose triacetate (CTA) membrane with embedded support (Fluid Technology Solutions, Albany, OR, USA) was used in this study. This membrane has been characterised and used by other researchers [11,35] in similar applications. The membrane came as a square sheet of dimensions 30.5 cm × 30.5 cm packed in 1% sodium metabisulfite water solution. Before use, the membrane was cut into the required dimensions of 9 cm × 25 cm (effective membrane area of 0.0225 m^2^) and thoroughly rinsed with deionised (DI) water. It was then soaked in DI water over night before use.

### 2.2. Feed and Draw Solutions

Feed samples were obtained from a local South African oil refinery effluent treatment plant (ETP) located in Durban in the KwaZulu-Natal province. The characteristics of the effluent were: SO_4_^2−^ = 855.756 ± 138.23 mg/L; CO_3_^2−^ = 306 ± 11.53 mg/L; pH = 9.09 ± 1.34 and conductivity 18.03 ± 4.38 mS/cm. Effluent characterisation was done according to procedures as used in our previous study [36]. Before the sampling point (three-phase separator), the effluent had already undergone preliminary treatment, where organic contaminants were removed and residual oils were recovered. The draw solution was prepared by dissolving 35 g/L of NaCl (Minema Chemicals (Pty), Johannesburg, South Africa) in distilled water (ELGA PURELAB Option-Q water deionizer, UK) to mimic the salinity of seawater.

### 2.3. Process Description

**Figure 1:**
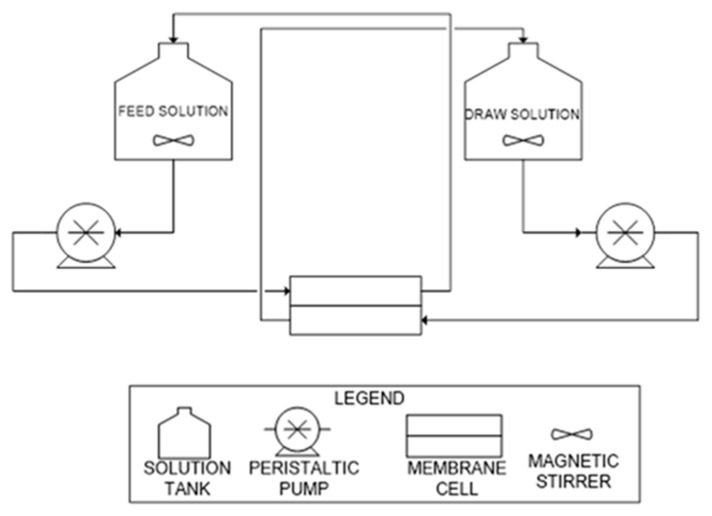
PFD of the FO process [36].

Figure 1 shows the process flow diagram (PFD) of this study, conducted in the counter-current flow mode, where the FS and DS enter and exit the membrane cell in opposite directions. A total of 5 experimental runs were conducted, each lasting for 6 h. The flow rate was kept at 9.4 L/h (maximum flowrate of pump) at both the feed and permeate sides of the membrane. Prior to the runs, pure water flux (PWF) of the virgin membrane was determined according to procedures as used by Cath, et al. [37].

Membrane cleaning was mainly by manual scrubbing and chemically enhanced osmotic backwash. During manual scrubbing, a brush was used to scour the surface of the membrane under running water for two minutes, allowing the bristles of the brush to dislodge foulants deposited on the membrane surface. This was performed after each run. The chemically enhanced backwash (performed after the fifth run) was achieved using 0.2% HCl (prescribed by manufacturer) in distilled water as the feed solution and 35 g/L NaCl as the DS, reversing the permeate flow. After membrane cleaning, PWF was determined to ascertain the degree of flux recovery. SEM analysis (Nova NanoSEM coupled with EDT and TLD detector, University of Cape Town, Cape Town, South Africa) was conducted for the membrane before its use, after manual scrubbing and after chemical cleaning. Equations (1)–(4) were utilized as follows:

(1) $$\mathrm{Permeate}\;\mathrm{flux}\;(J)=\frac{Volume\;of\;permeate\;\left(L\right)}{Effective\;membrane\;area\;\left(m^{2}\right)\times time\;\left(h\right)}$$

Volume of permeate was determined by taking the difference between the initial and final volumes of the draw solution.

Since the permeate diluted the *DS*, the dilution factor (*Df*) was calculated as follows:

(2) $$\mathrm{Dilution}\;\mathrm{factor}\;(\mathrm{Df})=\frac{V_{f,DS}}{V_{p}}$$

where *V_f_,_DS_* is the final volume of the *DS* and *V_p_* is the volume of permeate [11]:

(3) $$\mathrm{Component}\;\mathrm{Rejection}\;\mathrm{Efficiency}\;(\%)=\frac{C_{0}-D_{f}C_{f}}{C_{0}}\times 100$$

where *C*_0_ and *C_f_* are initial and final concentrations of the targeted component in the FS and *DS*, respectively and *Df* is the dilution factor [38]:

(4) $$\mathrm{WFR}\;\left(\%\right)\;=\;\frac{J_{c}}{J_{0}}\;\times \;100$$

where WFR is the water flux recovery. This measures the amount of pure water flux obtained after membrane cleaning, showing the efficiency of the cleaning process. $J_{c}$ = PWF (L/m^2^ h) after cleaning, $J_{0}$ = PWF of virgin membrane [39]:

(5) $$\mathrm{RSF}\;\left(g/m^{2}h\right)\;=\;\frac{C_{f}V_{f}-\;C_{0}V_{0}}{At}$$

where RSF is the reverse solute flux. This measures the amount of Cl^−^ that moved from the DS to the FS per unit area of membrane per hour.$\;C_{0}$ $\mathrm{and}\;C_{f}\;$ are initial and final concentrations (mg/L) of Cl^−^ in FS, respectively; $V_{0}$ and $V_{f}$ are initial and final volumes (L) of FS, respectively; A = membrane area (m^2^) and t = experimental time (h) [38].

## 3. Results and Discussion

### 3.1. Permeation Flux

#### 3.1.1. Water Flux and Water Flux Decline

Mass transport in FO encompasses both water transport and solute transport. This is driven by the DS concentration. Technically, as DS concentration increases, water flux and RSF also increases. In this study, a constant DS concentration of 35 g/L NaCl was used, generating approximately 29 bar osmotic pressure to drive mass transport [25].

**Figure 2:**
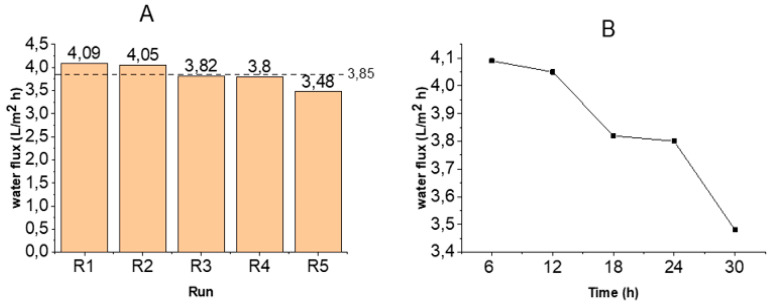
Graphs of water transport across the CTA membrane; (**A**): water flux and (**B**): trend of water flux decline with time.

Figure 2A shows the water flux for each run. The highest water flux obtained was 4.09 L/m^2^·h—this was obtained after the first 6 h of operation. Compared with the pure water flux, there was a 47.3% decrease in water flux. This decrease may be due to the reduction in the net osmotic pressure of the DS, caused by the cumulative effects of the intrinsic salinity of the FS and reverse salt diffusion as well as water transport.

As shown in Figure 2B, the water flux declined after each run. The trend of decline observed over the experimental period could be an indication of concentrative internal concentration polarisation (CICP). During CICP, water from the support layer, crossing the active layer, causes solutes within the interior surface of the active layer to become more concentrated. Consequently, the differential osmotic gradient across the active layer is reduced, hence leading to flux decline [20,30]. While this is expected, it is noteworthy that the rate of decline was low (cumulation of 15% flux decline for the entire duration of 30 h) compared to other cases reported in the literature. Li, et al. [40] reported a rapid decline in water flux during the dewatering of soluble algal products using NaCl as the draw solute. The decline was attributed to the combined effects of foulant accumulation and dilution of the DS. Additionally, Tang, et al. [41] reported a severe flux decline in their study of the coupled effects of ICP and fouling on flux behaviour in FO. The authors linked flux decline solely to the porosity of the support layer and the permeation of foulants into it. With the current study, the gradual flux decline could be due to the adoption of the manual scrubbing of the membrane after each run. Apart from preventing foulant build up on the membrane surface, the process may have dislodged foulants from within the pores of the membrane, which would have accumulated to cause severe fouling within the membrane structure.

#### 3.1.2. Reverse Solute Flux (RSF)

Reducing reverse solute flux remains one of the main challenges in FO applications. Generally, RSF is an indication of the concentration differential of draw solutes between the FS and DS. This difference forces draw solutes to move backwards from the DS to the FS until an equilibrium of solutes are established between the FS and the DS. This is undesirable, as it necessitates the replenishment of the draw solute as well as contamination of the FS [42,43].

**Figure 3:**
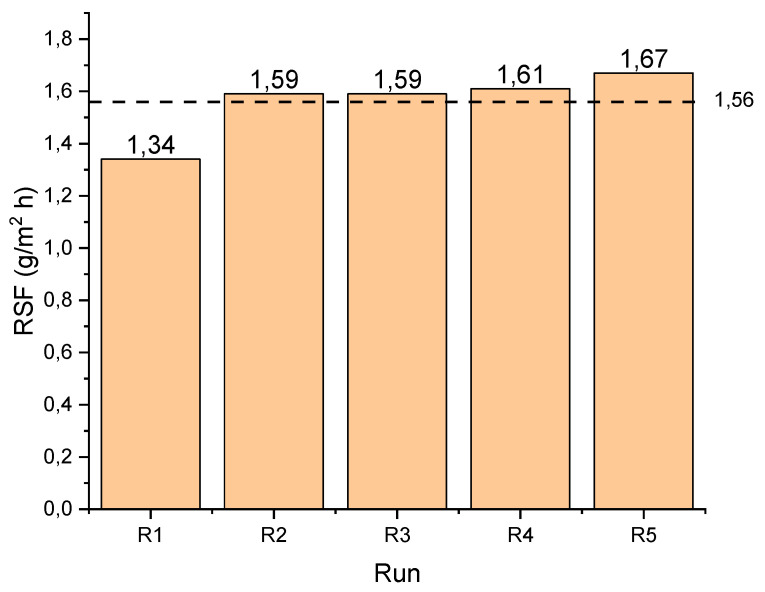
Reverse solute (Cl^−^) flux for each run.

Figure 3 displays the values of the RSF (Cl^−^ flux) for all runs, with an average RSF of 1.56 g/m^2^·h. The increase in RSF across the experimental duration may be due to increased deposits of Cl^−^ within the membrane pores, which later migrated into the FS due to the convective effects of the permeate water. Particularly with Cl^−^ being a univalent ion, this movement may be a lot easier to achieve.

In addition, research has shown that material deposition within the membrane structure is affected by cross flow of the bulk solution; as such, at the beginning of cross flow, there is little influence on the movement of ions [44]. This could be the reason for the increase between R1–R2 and the subsequent runs. Zhao, Zou and Mulcahy [23] reported slightly higher values for RSF in PRO mode under similar conditions using DI water as FS. In this study however, cognisance should be given to the fact that apart from the negative charge of the membrane’s active layer, which contributes to repulsion of the Cl^−^, the net Cl^−^ concentration differential between the FS and DS may be low due to the salinity of the FS, of which Cl^-^ may have been a part.

### 3.2. Salt Rejection Efficiency

**Figure 4:**
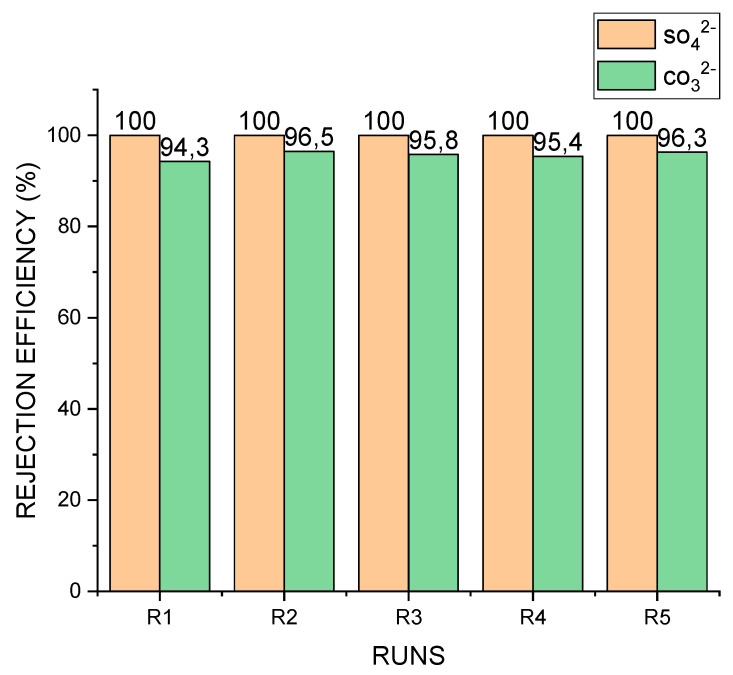
Rejection efficiency of SO_4_^2−^ and CO_3_^2−^. Average rejection efficiency of CO_3_^2−^ = 95.6%.

The membrane’s salt rejection efficiency is shown in Figure 4. It can be seen that for all 5 runs, SO_4_^2−^ rejection efficiency was 100%. This could be due to the coupled effects of the membrane properties and the properties of the sulphate ion, such as its divalent nature and relatively larger hydration radius of 0.379 nm [45]. In addition, SO_4_^2−^ is noted to have a low aqueous diffusion coefficient of 0.32 × 10^−5^ cm^2^/s [46], making its movement from one medium to another relatively slow. Consideration could also be given to the steric effect of the membrane and the multibarrier formed as a result of foulant deposition within the pores of the membrane.

A similar explanation could account for the efficient rejection of CO_3_^2−^. The average rejection efficiency of CO_3_^2−^ was 95.66 ± 0.32%. The divalent nature of CO_3_^2−^ and its aqueous diffusion coefficient of 0.92 × 10^−5^ cm^2^/s [47], as well as the properties of the membrane, may have contributed to the efficiency achieved—as in the case of SO_4_^2−^.

### 3.3. Fouling and Water Flux Recovery

The PRO mode is highly prone to membrane fouling due to the morphology of the porous support layer. The rough nature of the support layer promotes the deposition of foulants in micro recesses within the structure, making ICP and fouling pronounced. To add to this, studies have shown that molecules or ions with large sizes (like divalent and trivalent ions), lower aqueous diffusion coefficients and high viscosities, cause more pronounced ICP within the porous support layer of FO membranes [48]. Consequently, water flux declines. To determine the extent of water flux recovery, comparisons were made between the PWF of the virgin membrane (7.76 ± 0.12 L/m^2^·h) and the PWF of a used membrane after manual scrubbing and chemically enhanced osmotic backwash.

**Figure 5:**
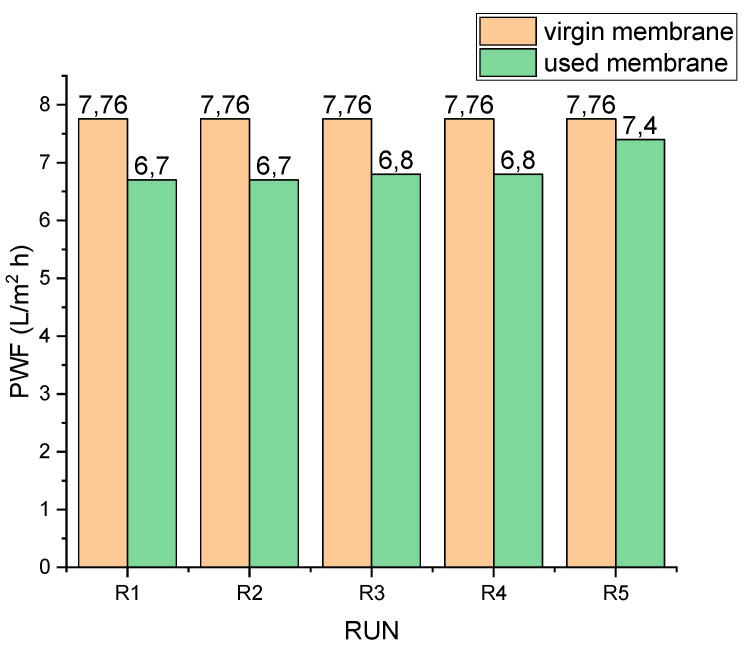
Water flux recovery: R1–R4 after manual scrubbing; R5 after chemically enhanced osmotic backwash.

Figure 5 shows the water flux recovery after each run. R1–R4 represent water flux recovery after manual scrubbing, while R5 shows the water flux recovery after chemically enhanced osmotic backwash. It can be seen that at least a flux of 6.70/7.76 L/m^2^·h was recovered, representing 86% of the PWF obtained by the virgin membrane. By implication, the majority of fouling was reversible; only 14% was irreversible. After the chemically enhanced osmotic backwash, up to 96% of flux was recovered. A similar study by Honda, et al. [30] reported a flux recovery of 70% after physical cleaning by crossflow flushing and 85% flux recovery after chemical cleaning. It appears that the use of manual scrubbing produced effective cleaning of the membrane, which further enhanced the chemical cleaning process.

#### 3.3.1. Effects of Manual Scrubbing

**Figure 6:**
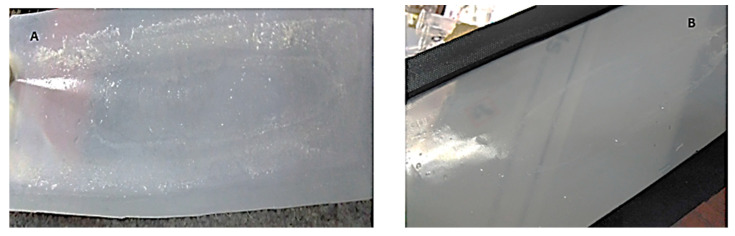
CTA membrane after experiment; (**A**): Before manual scrubbing; (**B**): after manual scrubbing.

Figure 6 shows the used membrane before and after manual scrubbing. In Figure 6A, deposits of salt precipitates and other contaminants can be seen on the membrane surface. This may have contributed to resistance to permeate flow across the membrane. Manual scrubbing under running water created the shear force-effect which dislodged the deposits from the membrane surface to remove the cake layer, hence paving the way for permeate flow. The manually cleaned membrane is shown in Figure 6B.

Pure water flux recovery after manual scrubbing of the membrane was 86% (on average, as shown in Figure 5; R1–R4). This is very significant, considering the fact that no chemicals were used. Again, flux decline between runs was minimal and gradual (Figure 2). Other studies [23,25] demonstrated a dramatic decline in flux in the PRO mode. This was attributed to foulant build up on the surface and within the porous structure of the membrane. It has, however, been demonstrated in this current study that constant physical scrubbing can drastically reduce the effects of foulant build up and hence its effects on flux decline. This is consistent with observations by She, et al. [49], who stated that foulant may only be deposited within the membrane support layer when its size is small enough to enter the support layer with the feed water convection. Consequently, there is a high possibility of foulant deposit on the membrane other than within the membrane structure.

#### 3.3.2. Effects of Chemically Enhanced Osmotic Backwash

**Figure 7:**
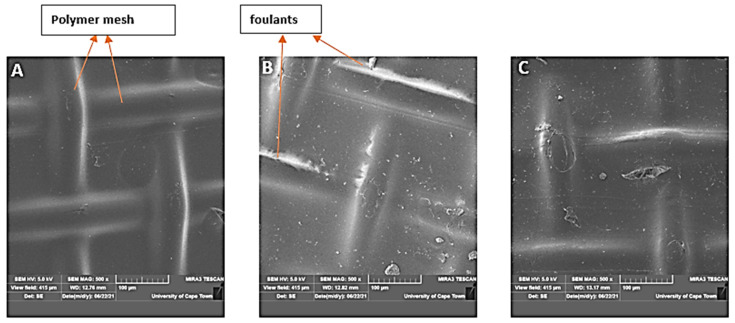
SEM micrographs of the porous support layer of CTA membrane—resolution of 500 µm, acceleration voltage of 5 kV, range of magnification = 10–50 k; (**A**): virgin membrane; (**B**): manually scrubbed membrane and (**C**): membrane after chemically enhanced osmotic backwash.

Figure 7 shows micrographs of scanning electron microscopy, performed for the CTA membrane before its use (virgin membrane; Figure 7A), after manual scrubbing (Figure 7B) and after chemically enhanced osmotic backwash (Figure 7C). Generally, osmotic backwash causes foulants within membrane pores to be dislodged into solution due to the reverse flow of the permeate. As permeate water is drawn in the opposite direction to the original flow, foulants that clog the membrane pores are forced off their positions and consequently swept into solution. As expected from Figure 7B, manual scrubbing was not sufficient in removing all foulants, since its effectiveness is limited to the immediate surface of the membrane.

Still on Figure 7B, it can be seen that majority of the foulants were deposited in the valley region close to the polymer mesh of the membrane which may be due to the local hydrodynamic conditions. Similar observations were made by Wang, et al. [24] in their study to develop a direct microscopic observation method for FO fouling characterization.

In Figure 7C, the SEM micrograph shows the condition of the membrane after osmotic back wash enhanced by 0.2% HCl solution. The choice of cleaning chemical was informed by the nature of fouling. Inorganic fouling was mainly expected due to the source of the feed. Rightly so (as shown in Figure 7B), crystalized particles were deposited within the membrane support structure. Water flux recovery after this cleaning session was 95% (as shown in Figure 5, R5). The effectiveness of osmotic backwash has been established in many previous studies, but mostly in the RO mode. Again, due to the fouling-resistant nature of the active layer (RO mode) of the membrane, chemicals are hardly used in osmotic backwash. However, in the PRO mode, the possibility of irreversible fouling is high [23].

To this effect, the efficiency of the osmotic backwash employed in this study can be linked to the following reasons: (1) the interaction of the cleaning chemical with the foulant and the membrane—HCl is well noted for the removal of inorganic foulants, dissolving foulants into solution or changing their nature to reduce their solubility or diffusivity; (2) the intermittent manual scrubbing of the membrane—manual scrubbing after every run minimized the accumulation of foulants on the membrane surface and in effect reduced the possibility of pore plugging by the foulants and increased thickness of the fouling layer within the membrane structure.

## 4. Conclusions, Implications and Recommendations

This study assessed the performance of FO in the PRO mode (where the active layer of the membrane faces the draw solution) during the desalination of a local oil refinery effluent. The assessment was made based on the permeation flux, salt rejection, membrane fouling and flux recovery after membrane cleaning. Membrane cleaning was achieved by adopting manual scrubbing of the membrane after each run and performing chemically enhanced osmotic backwash after the final run. The results showed an average water flux of 3.78 ± 0.13 L/m^2^ h, with a gradual decline in fluxes across all runs. Rejection of the target salts was 100% for SO_4_^2−^ and 95.66 ± 0.32% for CO_3_^2−^, which was mainly attributed to their divalent nature and aqueous diffusivities, as well as the properties of the membrane. RSF was 1.56 ± 0.11 g/m^2^·h. Manual scrubbing of the membrane after each run was identified as being very beneficial in maintaining the water flux across the runs, as well as in reducing the severity of ICP and membrane fouling, as shown by the SEM micrographs. Again, the chemically enhanced osmotic backwash was seen to be beneficial in flux recovery after the cumulative 30 h of experimental runs.

While the FO mode is mostly preferred in forward osmosis application, the following suggestions can be made based on the outcome of this study to improve FO application in the PRO mode: intermittent application of membrane scrubbing has the potential of reducing irreversible fouling within the membrane structure. This can be achieved in many ways, including the use of sponge balls or pneumatic cleaning, which introduces a shear force on the membrane surface, loosening and dislodging foulants. Ultimately, this reduces the frequency of osmotic backwash while maintaining water flux across the membrane.

For future studies, it is recommended that attention be paid to the development of more robust FO membranes with fouling-resistant support layers. The future of FO application is highly dependent on the membrane’s ability to resist fouling and the possibility of using both sides of the membrane with absolute ease.

## Figures and Tables

**Table 1:** Applications of FO for different orientations.

Membrane	FS	Membrane Orientation	DS	Results	Reference
Flat sheet CTA	Municipal wastewater: TSS = 400–800 mg/L, COD = 300–600 mg/L	FO mode	3.5 wt% NaCl solution	COD removal = 71.9%; Water flux = 3–7.4 L/m^2^·h:	[25]
		PRO mode		COD rejection = 69.7% Flux = 3–7.4 L/m^2^·h	
Flat sheet CTA	Activated sludge spiked with nutrients	FO mode	36 ± 1 g/L NaCl solution	Water flux = 5.62–6.25 L/m^2^·h; Nutrient rejection (NH_4_^+^-N PO_4_^3−^-P) > 96% and DOC rejection of 99%	[26]
Flat sheet CTA	Seawater	FO mode	6 M NH_4_HCO_3_	>95% rejection of salts	[27]
Flat sheet CTA	Deionized water	FO mode	1 M NaCl solution	Water flux = 10.39 L/m^2^·h, RSF = 0.084 mol NaCl/m^2^·h	[28]
Flat sheet CTA	26.1 mM CaCl_2_, 72 mM Na_2_SO_4_ and 10 mM NaCl	FO mode	Varied concentrations of NaCl	12% flux decline	[29]
		PRO mode		50% flux decline	

## Data Availability

Not applicable.
